# Intraosseous synovial sarcoma of the mandible: A case report and review of the literature

**DOI:** 10.3892/ol.2023.13904

**Published:** 2023-06-07

**Authors:** Ikumi Imajo, Tomohiro Yamada, Toru Chikui, Tamotsu Kiyoshima, Mamoru Ito, Kenichi Kohashi, Eiji Sakamoto, Yoshinao Oda

**Affiliations:** 1 Section of Oral and Maxillofacial Surgery, Division of Maxillofacial Diagnostic and Surgical Sciences, Faculty of Dental Science, Kyushu University, Higashi-ku, Fukuoka 812-8582, Japan; 2 Oral and Maxillofacial Radiology, Division of Maxillofacial Diagnostic and Surgical Sciences, Faculty of Dental Science, Kyushu University, Higashi-ku, Fukuoka 812-8582, Japan; 3 Laboratory of Oral Pathology, Division of Maxillofacial Diagnostic and Surgical Sciences, Faculty of Dental Science, Kyushu University, Higashi-ku, Fukuoka 812-8582, Japan; 4 Department of Hematology, Oncology and Cardiovascular Medicine, Kyushu University Hospital, Higashi-ku, Fukuoka 812-8582, Japan; 5 Department of Anatomic Pathology, Pathological Science, Graduate School of Medical Science, Kyushu University, Higashi-ku, Fukuoka 812-8582, Japan; 6 Section of Oral and Maxillofacial Oncology, Division of Maxillofacial Diagnostic and Surgical Sciences, Faculty of Dental Science, Kyushu University, Higashi-ku, Fukuoka 812-8582, Japan

**Keywords:** synovial sarcoma, oral, mandibular, intraosseous, extraosseous mass

## Abstract

Synovial sarcoma (SS) is a malignant soft tissue tumor that usually arises in the para-articular regions of the extremities. Only nine cases of SS in the mandible have been reported to date. The present study described a case of SS arising from the left mandible. A 54-year-old woman was referred to Kyushu University Hospital (Fukuoka, Japan) with a complaint of numbness in the left mental nerve area. Computed tomography revealed replacement of the left mandibular bone marrow with soft tissue and destruction of the mandibular canal. Magnetic resonance imaging revealed an isointense mass on T1-weighted images and hyperintensity on T2-weighted images. The tumor showed homogeneous enhancement. A biopsy was performed, and monophasic SS was diagnosed based on immunohistochemical staining features and genetic analysis. Hemimandible dissection and supraomophyoid neck resection were performed with fibular osteocutaneous flap reconstruction, followed by adjuvant chemotherapy. There was no evidence of recurrence or distant metastases. The present study also reviewed the clinical, imaging, histological, and immunohistochemical features of the SS in the mandible.

## Introduction

Synovial sarcoma (SS) is the fourth most common type of soft tissue sarcoma (STS) and accounts for 5–10% of all STS cases (1–3). SS most commonly arises in the deep soft tissue of the lower extremities, and ~7% of SS cases originate in the head and neck region, predominantly in the hypopharynx and parapharyngeal spaces (4,5). The term ‘synovial sarcoma’ was first proposed by Knox in 1936 because the tumor histologically resembled normal synovial tissue (6); however, this was a misnomer because SS does not originate from synovium. The widespread distribution of SS and the uncertain differentiation make the precise origin of SS still controversial; however, the prevalent onset in proximity of joints, bones, and skeletal muscles suggested a multipotent mesenchymal stem cell origin (7). STS occurring primarily within the bone is very rare (1,8,9), and only nine cases of SS in the mandible have been reported thus far (10–18).

As SS lacks characteristic symptoms and imaging findings, the clinical diagnosis of SS is often difficult. The present study presented an extremely rare case of monophasic SS arising from the mandibular bone marrow and described its clinical, imaging, histological, and immunohistochemical features.

## Case report

A 54-year-old woman was referred to our hospital with complaints of numbness and touch-evoked pain in the area innervated by left mental nerve area. The patient was otherwise healthy. She developed pain on mastication four months prior to the initial visit to Kyushu University Hospital (Fukuoka, Japan) and was diagnosed with temporomandibular joint disorder at a dental clinic and treated with an occlusal splint. However, the symptoms worsened with numbness appearing in the left chin. The patient was referred to an otolaryngologist, orthopedic surgeon, and neurosurgeon, but no abnormalities were noted. Therefore, she was referred to Kyushu University Hospital by the dental clinic for examination and treatment.

On initial examination, there was a slightly hard mass measuring 23×13 mm with spontaneous pain posterior to the left second molar. The mucosa overlying the mass was normal and non-adherent. Limited mouth opening limitation and enlargement of the regional lymph nodes were not observed. Neuropathy, such as allodynia and hypoesthesia, was observed objectively in the left mental nerve area. There was no medical history of induced neuropathy; therefore, radiological examinations were planned to determine the origin of the disease.

**Figure 1.**
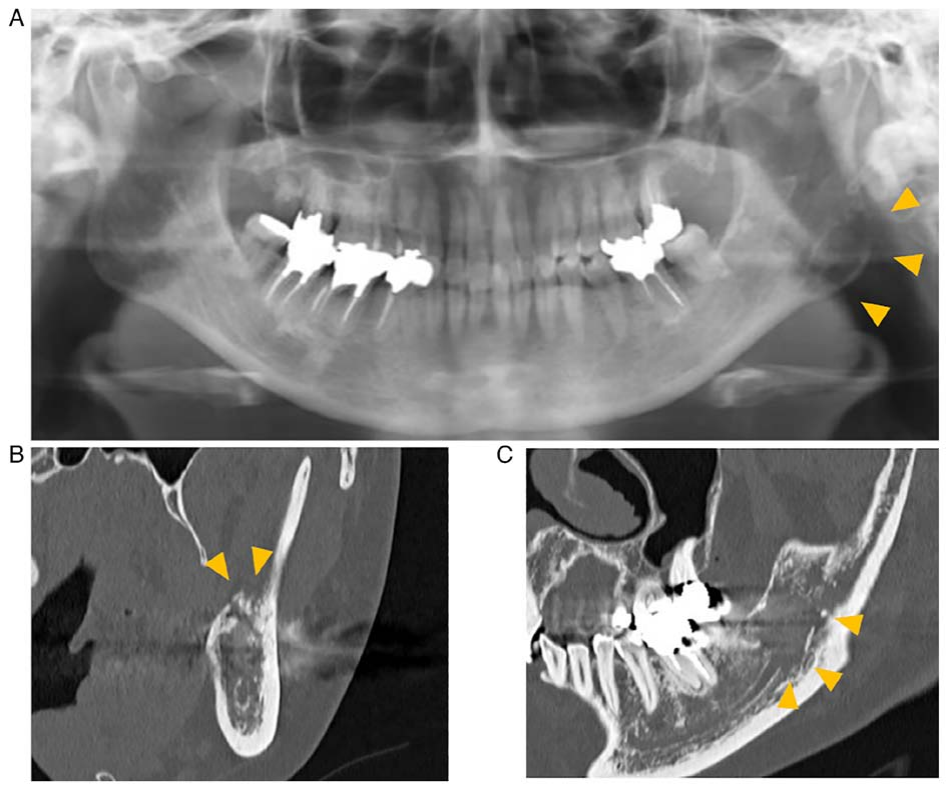
Panoramic and CT imaging findings. (A) Panoramic radiograph showing a radiolucent area (arrowheads) with ill-defined margins in the left mandibular angle to ramus region. (B) Coronal CT image showing small holes (arrowheads) in the cortical bone under the mass. (C) Sagittal CT image showing destruction of the mandibular canal (arrowheads). CT, computed tomography.

**Figure 2.**
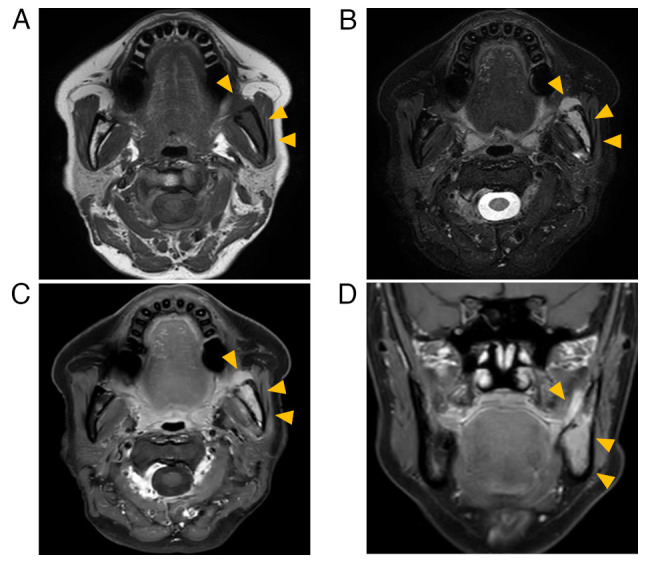
MRI findings. MRI showing (A) iso-intensity of a mass to muscle tissue (arrowheads) in a T1-weighted image and (B) hyperintensity (arrowheads) in a T2-weighted image. The tumor was homogeneously enhanced (arrowheads) using diethylenetriaminepentaacetic acid-gadolinium in (C) horizontal and (D) frontal image. The mass had an extraosseous extension. MRI, magnetic resonance imaging.

Panoramic radiography showed a poorly marginated radiolucent area in the left mandibular angle and ramus (Fig. 1A). Computed tomography (CT) revealed changes from marrow to soft tissue in the left mandible, small perforations in the cortical bone under the mass, and destruction of the left mandibular canal (Fig. 2B and C). Magnetic resonance imaging (MRI) revealed an isointense mass on T1-weighted images (T1-WI) and hyperintensity on T2-weighted images (T2-WI) in the bone marrow between the left lower molar region and the left mandibular notch. The mass had an extraosseous extension, and its maximum width and length were 55 and 25 mm, respectively. The tumor was homogeneously enhanced by gadobutrol (Gd-BTDO3A) (Fig. 2A-D). Positron emission tomography-CT showed mild fluorodeoxyglucose uptake in the extraosseous mass but no other lesions in distant organs. Blood biochemical test results did not reveal any abnormalities. The patient was clinically diagnosed with malignant lymphoma.

**Figure 3.**
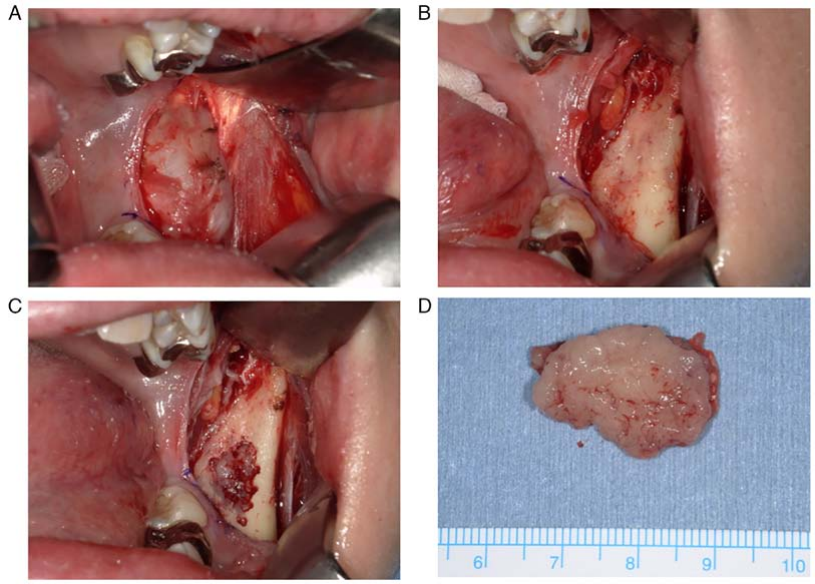
Intraoperative and gross tumor images. (A) The tumor was covered with periosteum. (B) The extraosseous mass peeled off easily from the mandible and there were small holes in the cortical bone that was in contact with the tumor. (C) A similar mass was also found in the mandible. (D) The tumor was fragile and yellowish white.

**Figure 4.**
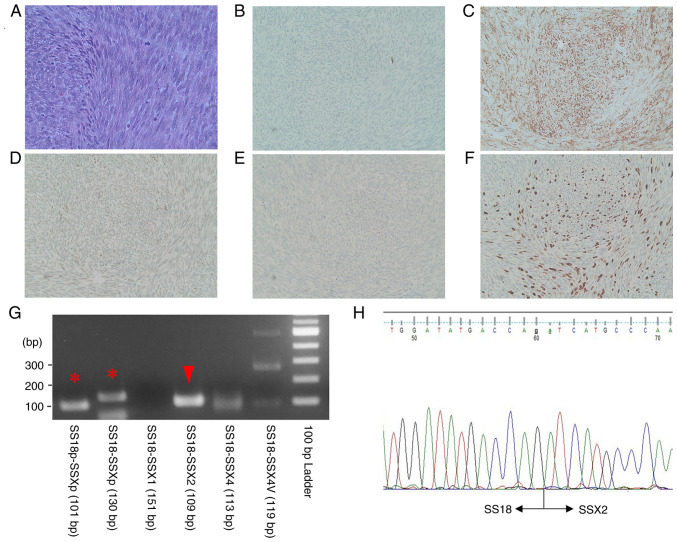
Histopathological and fusion gene findings. (A) The hematoxylin and eosin-stained section showed a proliferation of oval- to spindle-shaped cells that have hyperchromatic nuclei, arranged in a fascicular pattern (original magnification, ×400). Immunohistochemically, the tumor cells were positive for (B) AE1/AE3 (focal) and (C) SMA, and the expression of (D) SMARCB/INI1 was reduced compared with that in normal regions and (E) S-100 (original magnification, ×100). (F) The Ki-67 labeling index was 30% (original magnification, ×100). (G) The presence of the *SS18*-*SSX* fusion gene (asterisk) and *SS18*-*SSX2* fusion gene (arrowhead) were confirmed using PCR. (H) Targeted sequencing analysis identified *SS18*-*SSX2* fusions. SMA, smooth muscle actin. SMARCB/INI1, SWI/SNF related matrix associated actin dependent regulator of chromatin subfamily b/integrase interactor 1.

Biopsies were performed under general anesthesia. The possibility of solid tumors could not be completely ruled out, and tissue detachment was minimized to prevent dissemination. The extraosseous tumor lay under the periosteum; therefore, the tumor was removed along with the periosteum. The extraosseous tumor was easily detached from the mandible, and there were small holes in the cortical bone in contact with it. The mass was fragile and yellowish white. Intraosseous tissue was also collected from the bone marrow with small cortical bone removal because it could represent a different disease. Gross findings of the extraosseous and intraosseous tumors were the same (Fig. 3). The specimens were then subjected to histopathological examination. Imprint cytology was performed immediately, but malignant lymphoma was ruled out. Remaining specimens were fixed with 10% formalin neutral buffer solution for 24 h at room temperature. Fixed sections were embedded in paraffin and 4-µm-thick tissue sections were stained. Routine hematoxylin and eosin (H&E)-stained sections showed a dense proliferation of oval to spindle-shaped cells with hyperchromatic nuclei arranged in a fascicular pattern (Fig. 4A). Mitotic figures were frequently seen (15 mitoses in 10 high-power fields). Immunohistochemical staining except for SWI/SNF related matrix associated actin dependent regulator of chromatin subfamily b/integrase interactor 1 (SMARCB/INI1) were performed using a fully automated system [Leica Bond-III (Leica Microsystems GmbH) or VENTANA BenchMark ULTRA (Roche Applied Science)] and the following primary antibodies: Pan-cytokeratin (AE1/AE3; IS053; Dako; Agilent Technologies, Inc.), smooth muscle actin (SMA; cat. no. M0851; Dako; Agilent Technologies, Inc.), SMARCB/INI1 (cat. no. 612110; BD Biosciences), p16 (cat. no. 705-4713; Roche Diagnostics), CD34 (cat. no. NCL-L-END; Leica Microsystems GmbH), S-100 (cat. no. IR504; Dako; Agilent Technologies, Inc.) and Ki-67 (cat. no. M7240; Dako; Agilent Technologies, Inc.). The reaction of secondary antibody and following 3,3′-diaminobenzidine reaction were performed using EnVision+ System HRP Labelled Polymer kit (Dako; Agilent Technologies, Inc.). Appropriate positive control sections were mounted on the same slide glasses. Immunohistochemically, the tumor cells were positive for AE1/AE3 (focally; Fig. 4B) and SMA (Fig. 4C). The expression of SMARCB/INI1 was reduced compared with that in the normal region (Fig. 4D). The tumor was also positive for p16 but negative for CD34 and S-100 protein (Fig. 4E). The Ki-67 labeling index was 30% (Fig. 4F). The *SS18-SSX2* fusion gene was confirmed using PCR and sequencing analysis (Fig. 4G and H). RNA was extracted from formalin-fixed paraffin-embedded tissue using the RNAstorm kit (Cell Data Sciences). The reverse transcription was performed using RverTra Ace (Toyobo Life Science). PCR was performed using a KOD One (Toyobo Life Science) with SS18p-foward (5′-CCAGCAGAGGCCTTATGGATA-3′), SS18-foward (5′-GACCAACACAGCCTGGACCAC-3′), SSXp-reverse (5′-CGTTTTGTGGGCCAGATGCTTC-3′), SSX1-reverse (5′-GGTGCAGTTGTTTCCCATCG-3′), SSX2-reverse (5′-GCACTTCCTCCGAATCATTTC-3′), SSX4-reverse (5′-GCCTCTGGCACTTCCTTCAAAC-3′), SSX4V-reverse (5′-CGCTGATCTCTTCATAAACCAC-3′) primers. PCR conditions were initial denaturation at 95°C for 2 min, 45 cycles of 98°C for 10 sec, annealing at 64°C for 30 sec, 68°C for 20 sec, and a final extension 68°C for 5 min. PCR products were electrophoresed on 2.0% agarose gel and visualized using Midori Green Direct (NE-MG06; NIPPON Genetics). Sequencing was outsourced to another facility within the university. Sanger sequencing was performed using 3500×L Genetic Analyzer (Applied Biosystems; Thermo Fisher Scientific, Inc.). The tumor was diagnosed as SS, monophasic fibrous SS.

**Figure 5.**
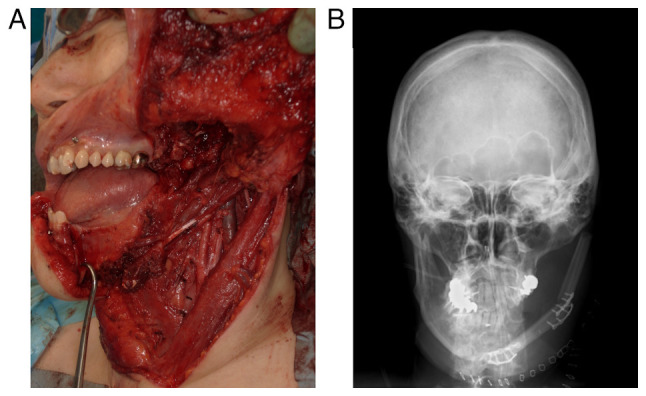
Intraoperative photograph and post-operative radiographical images. (A) Photograph taken after left supraomohyoid neck dissection and left hemimandibulectomy. (B) Post-operative radiographical image showing the mandible reconstructed by the fibula.

Multidisciplinary treatment was discussed with a medical oncologist, orthopedist, otorhinolaryngologist, and plastic surgeon. On imaging findings, the tumor seemed resectable, with a potential acceptable functional outcome after reconstruction; therefore, surgical treatment was decided upon. The patient underwent tracheotomy, left supraomohyoid neck dissection, left hemimandibulectomy, and immediate reconstruction using a fibular myocutaneous flap (Fig. 5). Histopathological examination revealed no cervical lymph node metastasis. Adjuvant chemotherapy with doxorubicin and ifosfamide was administered one month after surgery since SS arising in the head and neck has a high risk of distant metastases. After demineralization of the resected specimen, histopathological examination was performed. The SS penetrated the cortical bone and formed an extraosseous mass that invaded to the inferior alveolar nerve. Radiotherapy was not performed because the safety margins of the resected specimen were sufficient. At the 27-month follow-up, the patient was free of recurrence and metastasis.

## Discussion

SS occurring primarily within the mandible is very rare and only nine cases have been reported (10–18), the details of which are summarized in Table I. Variants of SS are classified into the monophasic, biphasic and poorly differentiated types (2,3). The monophasic type is subdivided into epithelial and fibrous type (11,14). Monophasic SS comprises spindle cells that are fairly uniform and relatively small, with sparse cytoplasm and ovoid, hyperchromatic nuclei with granular chromatin and inconspicuous nucleoli. Biphasic SS has epithelial and spindle cell components in varying proportions. The epithelial cells are arranged in solid nests or cords, or in glands with a tubular or occasionally alveolar or papillary architecture. The spindle cells in biphasic SS resemble the spindle sells found in monophasic SS. In otherwise monophasic or biphasic SS, poorly differentiated areas with increased cellularity, greater nuclear atypia, and high mitotic activity may be found (5). SS is characterized by a specific chromosomal translocation t(X;18)(p11;q11) (5). This translocation leads to the formation of a SS18-SSX fusion protein, which drive sarcomagenesis (2,7,19). The fusion protein integrates, by means of the SS18 component, into barrier-to-autointegration factor (BAF; also known as mammalian SWI/SNF) family complexes, which have crucial roles in chromatin organization (7). The SS18-SSX fusion protein, by inducing imbalance in BAF family complexes, can alter chromatin remodeling and activate aberrant gene transcription (7). The SSX component mediates interaction with polycomb chromatin repressor complexes involved in gene transcription inhibition. By inducing broad transcriptional dysregulation, the *SS18-SSX* fusion oncogene represents a major driver of transformation and malignancy (7). The *SS18-SSX* fusion gene has subtypes, including *SS18-SSX1, SS18-SSX2* and *SS18-SSX4* (2,5,7). The subtype of fusion gene correlates with the tumor phenotype; almost all biphasic SS has been shown to harbor the *SS18-SSX1* fusion gene, and almost all of the *SS18-SSX2* tumor are monophasic SS (2,3,7,19).

The clinical appearance and symptoms of head and neck SS vary among the reported cases and are usually determined by the tumor site (3,4). SS arising from the oral cavity mainly presents as a slowly enlarging, painless, non-tender, spherical, and deeply seated mass (4,11,14). Patients become symptomatic when the size of the SS grows enough to cause pressure symptoms on adjacent structures (4). In the present patient, the cause of the pain was thought to be inferior alveolar nerve compression by the tumor because the pain decreased soon after cortical bone opening by incisional biopsy. However, the hypoesthesia of the left chin did not improve. Therefore, the cause of the hypoesthesia was thought to be invasion of SS into the inferior alveolar nerve.

On CT, SS presents as a uniform and well-defined lesion (4,8,9). On MRI, SS displays an image that has been described as a triple signal pattern, which reflects a combination of calcification, cystic changes due to necrosis and hemorrhage and air-fluid levels (2,8,9,20). In the case of intraosseous SS, lesions appear osteolytic on plain radiography, low- or iso-intense on T1-WI MRI, of variable intensity on T2-WI MRI, and heterogeneously enhanced using diethylenetriaminepentaacetic acid-gadolinium (8). There are no characteristic imaging findings for SS; therefore, it is difficult to diagnose it using only imaging modalities. SS originating from the bone forms an extraosseous mass via the Haversian system, similar to that seen in the current case (8,9,20). The formation of an extraosseous mass without bone destruction is a rare feature of other solid tumors; therefore, it may be a characteristic finding of intraosseous SS.

Biopsy is essential for proper treatment planning. Options for biopsy include incisional biopsies, core needle biopsies (CNB) and fine needle aspirations (FNA). CNB and FNA guided by imaging are useful for deep-seated tumors, but they tend to have lower diagnostic accuracy than do open incisional biopsy because they cannot provide large tissue samples (2). CNB and FNA are also associated with the risk of dissemination owing to needle tract seeding. Biopsy should be performed properly according to the location and size of the tumor. Since the tumor in the present case was under the oral mucosa and not deep, an open incisional biopsy was performed. The incision was designed to be included on the resection side and tissue detachment was minimized to prevent dissemination, and large tissue samples were taken from both extraosseous and intraosseous tumor to make a definitive diagnosis. As the extra- and intraosseous tumors were the same, biopsy of intraosseous tumors was unnecessary. In cases of SS, it is harder to microscopically diagnose monophasic types than biphasic types, particularly in unusual locations because of the resemblance to fibrosarcoma or other spindle cell tumors (14,17,18). In the jaws, additional consideration to other odontogenic spindle cell tumors is required (17). Therefore, in addition to routine H&E staining, immunohistochemical staining is also available to facilitate diagnosis. SMARCB/INI1 expression is downregulated in SS and the same finding was noted in the present case (21). SMARCB/INI1 is also known as BAF47. The SS18-SSX fusion proteins competitively replace the wild-type SS18 in canonical BAF complex, thus resulting in ejection of SMARCB/INI1 (22). The presence of the *SS18-SSX* fusion gene confirms the diagnosis in difficult cases with unusual histological features or unusual locations (4,13,15,16,20,23).

Owing to the paucity of SS cases in the oral and maxillofacial areas, information regarding appropriate therapy is limited. Surgical resection is the mainstay of therapy for localized STS, and the adequate margin size depends on several factors (24). For STS, a margin of ≥1 cm or an intact anatomic barrier is recommended, and the same is true for SS (24). Radical excision with negative margins is most important for local control and overall survival of SS patients (2). However, radical excision with negative margins is not always possible in the head and neck region because of the complicated anatomy, and radiation therapy is often recommended (4). Adjuvant radiation therapy improves local control of head and neck SS (4). Preoperative radiation is associated with an increase in wound complication rate, while post-operative radiation can cause fibrous and joint stiffness, which may lead to long term dysfunction (2). Unlike the majority of STS, SS appears to be more chemosensitive (2,7). However, adjuvant chemotherapy remains controversial since the results of randomized trials are non-conclusive (2,7). In general, chemotherapy including anthracyclines and ifosfamide is administered for high-risk or advanced patients (2,3,7,24). Treatment options should be decided by a multidisciplinary team based on the patient's age, performance status, comorbidities, tumor location, and histological subtype. In the present case, neoadjuvant radiation therapy was considered ineffective because the SS was mostly surrounded by thick cortical bone, and the safety margin was sufficient for histopathological examination; therefore, adjuvant radiation therapy was not performed. Considering the high rate of distant metastasis in patients with SS, systemic chemotherapy with doxorubicin and ifosfamide was administered. Neoadjuvant chemotherapy effect can be assessed by determining the changes in tumor size; however, if it is not effective and progresses to the skull base, the tumor becomes unresectable. So, chemotherapy was administered post-operatively in the present case.

The prognosis of SS is affected by tumor size, location, patient age, extent, histological subtype, mitotic activity, fusion type, margin of resection and adjuvant radiotherapy (1–4,12–15,20,23). The 5-year survival rate of patients with SS originating in the jaw is 69.1% (1). Late local recurrences and pulmonary metastasis >5 years after the initial diagnosis are more typical of SS than other sarcomas (2–4,7,16,20). Long-term follow-up is necessary because the prognosis is often poor, and a number of patients develop lung metastasis.

The present study was a report of a rare case of SS arising in the left mandible. Intraosseous SS penetrated via the Haversian system and spread outside the bone. There are no characteristic imaging findings for SS, but the formation of an extraosseous mass without bone destruction may be a characteristic finding of intraosseous SS.

## Figures and Tables

**Table I.** Summary of clinical features of nine previously reported cases and present case of SS arising from the mandible.

First author, year	Age, years	Sex	Site	Symptoms	Subtype	Treatment	Outcome	(Refs.)
Torsiglieri, 1991	28	Male	Body	Swelling	UK	S, C, R	Dead (3 y 8 m)	(10)
Koga, 2005	42	Male	Body	Swelling	UK	S	Alive (7 y)	(11)
Granowetter, 2006	11	Male	UK	Pain, trismus	UK	C, R, S	Alive	(12)
Tilakaratne, 2006	29	Female	Condyle	Swelling	UK	S, R	Alive (2 y)	(13)
Wang, 2008	32	Male	Condyle	Swelling, trismus	B	S	UK	(14)
Tao, 2011	20	Female	Body	Swelling	M	S, R	Alive (1 y)	(15)
Wadhwan, 2011	28	Male	Body	Swelling, pus discharge, pain	B	S	Alive (1 y)	(16)
Khalili, 2012	76	Male	Body	Swelling, pain, paresthesia	M	S	Dead (2 m)	(17)
Teixeira, 2021	22	Male	Body	Pain, swelling	M	UK	Alive (2 y)	(18)
Imajo, 2023	54	Female	Body	Pain, paresthesia	M	S, C	Alive (27 m)	Present case

B, biphasic; M, monophasic; S, surgery; C, chemotherapy; m, months; y, years; R, radiation therapy; UK, unknown.

## Data Availability

All data generated or analyzed during this study are included in this published article.

## Abbreviations

SS: synovial sarcoma
STS: soft tissue sarcoma
CT: computed tomography
MRI: magnetic resonance imaging
T1-WI: T1-weighted images
T2-WI: T2-weighted images
H&E: hematoxylin and eosin
SMA: smooth muscle actin
CNB: core needle biopsy
FNA: fine needle aspiration
SMARCB/INI1: SWI/SNF related matrix associated actin dependent regulator of chromatin subfamily b/Integrase interactor 1
